# Evaluation of a pressure plate for detection of hind limb lameness in cats

**DOI:** 10.1371/journal.pone.0231904

**Published:** 2020-04-22

**Authors:** Eva Schnabl-Feichter, Alexander Tichy, Barbara Bockstahler

**Affiliations:** 1 Department of Companion Animals and Horses, University Clinic for Small Animals, Small Animal Surgery, Section of Physical Therapy, University of Veterinary Medicine, Vienna, Austria; 2 Department of Biomedical Sciences, Platform of Bioinformatics and Biostatistics, University of Veterinary Medicine, Vienna, Austria; Faculty of Animal Sciences and Food Engineering, University of São Paulo, BRAZIL

## Abstract

Detection of lameness in cats can be very time-consuming and frustrating. Feline studies have shown that the success of treatment can be evaluated by measurement of the ground reaction force (GRF). However, the possibility of multiple limb involvement or the presence of a compensatory mechanism has not been investigated. Furthermore, there has been no research in cats on possible differences in GRFs between those with stifle problems and those with hip problems, as reported in dogs. In this study, we compared temporospatial parameters and GRFs in 20 lame cats after femoral head and neck ostectomy (FHO) or stifle disease to those in 15 healthy cats. An orthopedic examination was performed in all cats and radiographs were obtained to confirm the disease. GRFs, including peak vertical force (PFz), vertical impulse (IFz), time to PFz, and temporospatial parameters, including step length, paw contact area, and stance phase duration, were calculated. We also calculated the symmetry index (SI) in the forelimbs and hind limbs. The GRFs were normalized to total force (% TF). We found that the IFz (% TF) and PFz (% TF) were lower in the affected limb than in the other limbs in the lame cats. When the lame cats were compared with the sound cats, this difference was only significant for IFz (% TF). The SI values for the PFz and IFz were significantly higher in the hind limbs than in the forelimbs in the lame cats group but there was no difference in the SI according to whether the problem was in the hip or stifle. There were also differences in stance phase duration and paw contact area in both the forelimbs and hind limbs between the sound group and the lame group. There was no difference in PF_Z_ (% TF) or IF_Z_ (% TF) in the affected limb between the lame cats with stifle and those after FHO; however, there were changes in time to PFz and step length. In conclusion, mild to moderate lameness can be detected and measured in cats using pressure plates. The compensatory mechanisms in cats at a walk appear to involve shifting the weight to the other three legs without favoring either the contralateral or the diagonal limb.

## Introduction

Several methods have been developed to differentiate a normal gait from an abnormal one [1]. However, these methods tend to be subjective, e.g., direct visual observation of gait during locomotion [2, 3]. Assessment of gait is particularly challenging in cats because they do not like to move around in unfamiliar surroundings and need time to acclimatize. Furthermore, cats often adopt a crouched position in the consulting room, making it even more difficult to evaluate gait [4, 5]. Much effort has been made to establish objective outcome measurements in cats [6–11]. The main advantages of measuring ground reaction force (GRF) are less observer bias (meaning less subjectivity) and the ability to collect and store data [9].

In recent years, there has been increasing interest in gait analysis in cats [6–11]. Pressure plates are often used to measure temporospatial parameters and GRFs, including PFz (peak vertical force) and IFz (vertical impulse). These measurements have been confirmed to be reliable and comparable from breed to breed [10, 11]. Measurement of GRFs (PFz and IFz) can detect lameness in dogs and cats [12–15]. Furthermore, the symmetry index (SI), which assesses the degree of symmetry between the forelimbs and hind limbs, can be used to determine whether an animal is clinically sound or lame [12–15].

Historically, lameness was considered uncommon in cats and a rare reason for a visit to a veterinary hospital [4, 5]. However even if pathological changes on radiographs are common in cats [16–19], it is an infrequent cause of lameness [17]. Whether or not there is multiple limb involvement or a compensatory mechanism in cats is unknown. Objective gait analysis has been used to investigate osteoarthritis [12, 13, 20] and pain [21] in cats. There has been research on the effects of cranial cruciate disease in cats both experimentally [22] and in vivo [23] as well as a case report on the effects of bilateral FHO in a cat [24]. However, to our knowledge, there have been no studies of lameness or possible compensatory mechanisms in cats.

The aims of the study were to determine the ability of a pressure-sensitive mat to detect hind limb lameness in cats at a walk, differentiate their gait from that of sound cats, confirm if there is any compensatory distribution of force (e.g. to the contralateral hind limb or to one of the forelimbs), and determine if differences in GRFs and temporospatial parameters could be detected in cats after femoral head and neck ostectomy (FHO) or stifle joint disease. We hypothesized that lame cats would have lower PFz and IFz values in the affected hind limb and that the compensation would be in the contralateral hind limb and not in the diagonal forelimb. We also hypothesized that the GRFs would be significant lower in cats with stifle joint disease than in cats with lameness after FHO.

## Material and methods

### Animals

This non-randomized prospective study was approved by the Institutional Ethics and Animal Welfare Committee of the University of Veterinary Medicine Vienna/Austria and was performed in accordance with Good Scientific Practice guidelines and national legislation (reference number 13/10/2017). All owners who participated with their cats in the study signed a written consent.

The study included 20 client-owned cats with previously (> 1 year ago) performed unilateral femoral head and neck ostectomy (n = 13) or stifle (n = 7) joint disease and lameness that could be detected by a board-certified surgeon (ESF) either on orthopedic examination or from pressure plate measurements (LC group). The cats in this group where otherwise healthy and did not have any other neurologic or orthopedic disease. This group consisted of fifteen domestic shorthaired cats, two Maine Coons, one Persian, one British Shorthair, and one domestic longhaired cat. All clinical, neurological, and orthopedic examinations were performed by the same surgeon (ESF). Radiographs of the hip and stifle joints were obtained during the initial examination. None of the cats showed high-grade lameness, such as non-weight-bearing or toe-touching only (4/5 or 5/5). The LC group included two intact males, fifteen neutered males, and three spayed females of mean age 7.8 ± 3.9 (range, 1.8–16.0) years. Further descriptive data for the LC group are shown in Table 1.

**Table 1:** Characteristics of the 20 lame cats in this study.

No	Breed	Sex	Age (mo)	BW (kg)	Lameness grade (1–5)	Leg	Joint	Cause/Surgery
**1**	DSH	FC	129	5	2	Left	Stifle	CCLR/lateral suture
**2**	DSH	MC	22	5.9	2	Left	Stifle	Arthritis
**3**	DSH	MC	21	4.5	2	Left	Stifle	Patella luxation
**4**	DSH	FC	155	4.6	3	Right	Stifle	Gonarthrosis
**5**	BSH	MC	157	3.8	2	Right	Stifle	Gonarthrosis
**6**	DLH	MC	104	5.6	2	Left	Stifle	Gonarthrosis, old tibial fracture
**7**	Maine Coon	M	57	5.3	3	Right	Stifle	Patella luxation
**8**	DSH	MC	54	8	0	Right	Hip	Femoral neck fracture/FHO
**9**	DSH	MC	88	4.8	1	Left	Hip	Hip luxation /FHO
**10**	DSH	M	115	5.6	1	Right	Hip	Femoral head fracture/FHO
**11**	DSH	MC	113	6.1	1	Right	Hip	Hip luxation/FHO
**12**	DSH	MC	98	5.9	3	Left	Hip	Chronic slipped capital physis/FHO
**13**	Persian	MC	76	4.9	1	Left	Hip	Slipped capital physis/FHO
**14**	Maine Coon	MC	118	8.3	1	Left	Hip	Slipped capital physis/FHO
**15**	DSH	MC	114	6.4	0	Left	Hip	Hip luxation /FHO
**16**	DSH	MC	24	4.65	0	Right	Hip	Hip luxation /FHO
**17**	DSH	FC	192	2.8	1	Left	Hip	Hip luxation /FHO
**18**	DSH	MC	104	5.6	1	Left	Hip	Hip luxation /FHO
**19**	DSH	MC	50	4.8	0	Right	Hip	Chronic femoral neck fracture/FHO
**20**	DSH	MC	58	5.5	0	Right	Hip	Hip luxation /FHO

BSH = British Shorthair, BW = body weight, CCLR = cranial cruciate ligament rupture, DLH = Domestic Longhair, DSH = Domestic Shorthair, FC = female castrated, FHO = femoral head and neck ostectomy, M = male, MC = male castrated, mo = month.

The data collected for the LC group were compared with those for 15 client-owned domestic shorthaired control cats (NLC group) that had been investigated in a previous study [8, 9]. These cats had been free of any lameness and had normal clinical, orthopedic, and neurological findings with no radiographic signs of pathology. This group consisted of seven neutered males and eight spayed females with a mean age of 7.2 ± 4.2 (range, 2.6–14.9) years.

### Experimental protocol and equipment

All clinical and radiographic examinations and pressure plate measurements were performed at the University of Veterinary Medicine in Vienna. The gait measurements in the LC group were performed on initial presentation in the same dedicated quiet room with the owner and two researchers present, as in the NLC group. The data were collected using a 203.2-cm × 54.2-cm Zebris FDM Type 2 pressure plate (Zebris Medical GmbH, Allgäu, Germany), which contains 15,360 sensors, has a sampling rate of 100 Hz, and is mounted in the middle of a 7-m runway. This pressure-sensitive plate was covered with a rubber mat to prevent slipping and remain hidden from the cat. All measurements were video-recorded using a Panasonic NV-MX500 camera. The data obtained were stored using WinFDM software (v1.2.2; Zebris Medical) and processed using specially developed software (Pressure Analyzer 1.3.0.2; Michael Schwanda).

After a few minutes of acclimatization in the gait analysis room, the cat was encouraged with toys, food, and verbal and visual stimuli to cross the pressure-sensitive plate in a straight line, sometimes with a portable cartoon wall placed on one side of the walkway. Gait cycles were excluded when there was an apparent change in velocity (trotting or stopping), when the cat left the plate, or when it turned is head. As described previously [10, 11], a measurement was deemed to be valid if the cat crossed the plate at least three times, allowing for at least five valid step cycles to be measured.

### Data processing and outcome parameters

Gait velocity was recorded from the left forelimb. The gait parameters evaluated were PFz (N), IFz (N), time to PFz (% StPh), step length (m), paw contact area (cm^2^) and stance phase duration (SPD, seconds). The GRF data were normalized to percent total force (% TF) as recommended by other investigators [10, 25]. The symmetry index (SI %) was calculated from the PFz and IFz for the forelimbs and hind limbs.

The SI for the contralateral limb pair was calculated using the formula [10, 11]:

$$SIXFz=abs\left(\frac{\left(XFzFL-XFzFR\right)}{\left(XFzFL+XFzFR\right)}\right)\times 100$$

where, SI is the symmetry index, X is the given value for PFz or IFz, abs is the absolute number, FL is the left forelimb, and FR is the right forelimb. Hind limb symmetry was calculated so that an SI of 0% would represent perfect symmetry between the contralateral limb pair and therefore would mean no lameness.

All lame legs, regardless of which side, were assigned to be the left hind limb for more detailed comparison between the two study groups and the different limbs. Accordingly, the right hind limb was always the contralateral limb, the left forelimb was always the ipsilateral limb, and the right forelimb was the diagonal limb.

### Statistical analysis

The data for the NLC group were obtained in a previous study [10, 11]. The Kolmogorov-Smirnov test was used to analyse the assumption of normal distribution of the parameters in the LC group. Descriptive statistics were calculated for each parameter. The data are presented as the mean and standard deviation. A general linear model with repeated measures was used to compare the lame, contralateral, ipsilateral, and diagonal limbs between the LC and NLC groups. Each parameter was compared using an independent-samples *t*-test to detect statistically significant differences between the LC and NLC group and between stifle and hip disease in the LC group. We used Sidak´s alpha correction procedure to avoid alpha accumulation due to multiple testing. All statistical analyses were performed using SPSS statistical software version 24 (IBM Corp., Armonk, NY, USA). A *p*-value < 0.05 was considered statistically significant.

## Results

All data were normally distributed. There was no significant difference in the body weight between the LC group and NLC group (4.9 ± 1.3 [3.2–8.1] kg vs. 5.0 ± 1.1 [8–6.6] kg or in mean gait velocity (0.58 ± 0.14 [0.30–0.85] m/s vs. 0.70 ± 0.09 [0.52–0.83]).

### Ground reaction forces

The GRF and temporospatial parameters measured in the LC and NLC groups are summarized in Table 2 while the measurements for cats with hip disease and those with stifle disease in the LC group are compared in Table 3.

**Table 2:** Ground reaction forces (normalized to %TF) and temporospatial parameters of 20 lame cats and 15 sound cats.

		PFz, % TF	TPFz, % StPh	IFz, % TF	SPD, s	SL, m	PCA, cm^2^
**Forelimb, left/IPS**	LC	29.49 ± 1.38^2^^,^^3^ (27.54–32.42)	62.46 ± 5.83^2^^,^ ^3^ (52.78–76.36)	30.04 ± 2.08 ^2^^,^^3^ (27.49–35.54)	0.53 ± 0.11^2^^,^* (0.38–0.79)	0.48 ± 0.05 (0.39–0.57)	13.30 ± 1.58^2^^,^^3^ (10.76–17.45)
	NLC	29.12 ± 2.55^2^^,^ ^3^ (25.37–34.82)	58.36 ± 7.35^2^^,^ ^3^ (45.99–67.24)	29.23 ± 2.53^2^^,^ ^3^ (26.05–35.20)	0.45 ± 0.07^2^^,^ ^3^^,^* (0.35–0.57)	0.50 ± 0.04 (0.41–0.56)	12.58 ± 1.35 (10.42–15.24)
**Forelimb, right/DI**	LC	29.78 ± 1.33^2^^,^^3^ (27.39–31.72)	63.84 ± 5.35^2^^,^ ^3^^,^* (54.55–72.64)	30.53 ± 2.20 ^2^^,^^3^ (28.26–34.71)	0.54 ± 0.11^2^^,^ ^3^* (0.39–0.75)	0.49 ± 0.05 (0.40–0.64)	13.20 ± 1.79^2^^,^^3^ (10.50–18.01)
	NLC	28.98 ± 2.33^2^^,^ ^3^ (26.13–35.54)	58.80 ± 5.65^2^^,^ ^3^^,^* (48.25–68.59)	29.29 ± 2.42^2^^,^ ^3^ (25.75–34.61)	0.45 ± 0.06^2^^,^ ^3^^,^* (0.34–0.56)	0.49 ± 0.05 (0.40–0.64)	12.79 ± 1.59 (10.40–15.78)
**Hind limb, left/L**	LC	19.90 ± 1.34^1^^,^ ^2^^,^ ^3^ (17.45–22.41)	48.65 ± 7.47^2^^,^ ^3^ (36.90–61.48)	17.99 ± 1.72 ^1^^,^ ^2^^,^ ^3^ (13.48–20.87)	0.46 ± 0.10^1^^,^ ^2^^,^ ^3^ (0.30–0.66)	0.49 ± 0.05 (0.38–0.58)	12.30 ± 1.93^2^^,^^3^ (9.52–16.97)
	NLC	20.83 ± 2.32^2^^,^ ^3^ (15.73–24.49)	47.91 ± 10.43^2^^,^ ^3^ (27.76–67.77)	20.60 ± 2.45^2^^,^ ^3^ (15.26–24.06)	0.43 ± 0.06^2^^,^ ^3^ (0.33–0.54)	0.51 ± 0.05 (0.43–0.60)	12.27 ± 2.27 (9.33–16.37)
**Hind limb, right/CO**	LC	20.89 ± 1.49^1^^,^ ^2^^,^ ^3^ (18.10–23.38)	46.49 ± 7.78^2^^,^ ^3^ (35.48–58.71)	21.44 ± 1.73 ^1^^,^ ^2^^,^ ^3^ (18.13–25.32)	0.51 ± 0.11^1^^,^ ^2^^,^* (0.36–0.72)	0.48 ± 0.05 (0.39–0.57)	12.49 ± 1.95^2^^,^^3^ (9.86–17.48)
	NLC	21.07 ± 2.67^2^^,^ ^3^ (13.91–24.22)	45.17 ± 9.51^2^^,^ ^3^ (34.96–60.94)	20.88 ± 2.49^2^^,^ ^3^ (14.93–24.06)	0.43 ± 0.06^2^^,^ ^3^^,^* (0.34–0.53)	0.51 ± 0.06 (0.42–0.61)	12.30 ± 2.25 (8.53–16.14)

CO = contralateral, DI = diagonal, IFz = vertical impulse, IPS = ipsilateral, L = lame, LC = lame cats, NLC = sound cats, PCA = paw contact area, PFz = peak vertical force, SL = step length, SPD = stance phase duration, StPh = stance phase, TF = total force, TPFz = time to PFz. The grey shaded area indicates the lame leg.

^1^Significant difference between contralateral limb pair,

^2^significant differences between ipsilateral limb pair,

^3^significant differences between diagonal limb pair,

*significant differences for the limb between lame and sound cats.

**Table 3:** Ground reaction forces (normalized to %TF) and temporospatial parameters in 7 cats with stifle joint disease and 13 after FHO.

		PFz, % TF	TPFz, % StPh	IFz, % TF	SPD, s	SL, m	PCA, cm^2^
**Forelimb, left/IPS**	SJD	29.28 ± 1.99 (27.54–32.42)	61.58 ± 4.36 (57.21–69.26)	29.99 ± 3.12 (27.49–35.54)	0.51 ± 0.09 (0.42–0.68)	0.45 ± 0.05 ^a^ (0.39–0.53)	12.85 ± 1.05 (11.46–14.52)
	FHO	29.60 ± 0.99 (27.70–30.88)	62.93 ± 6.00 (52.78–76.36)	30.07 ± 1.42 (27.79–32.25)	0.54 ± 0.13 (0.38–0.79)	0.50 ± 0.04 ^a^ (0.42–0.57)	13.54 ± 1.79 (10.76–17.45)
**Forelimb, right/DI**	SJD	29.43 ± 1.35 (28.35–31.72)	64.48 ± 6.02 (54.55–71.30)	30.29 ± 1.78 (28.73–32.85)	0.51 ± 0.09 (0.43–0.67)	0.45 ± 0.05 ^a^ (0.40–0.51)	12.61 ± 1.23 (11.23–14.79)
	FHO	29.97 ± 1.34 (27.39–31.67)	63.50 ± 5.18 (55.57–72.64)	30.66 ± 2.45 (28.26–34.71)	0.55 ± 0.12 (0.39–0.75)	0.51 ± 0.04 ^a^ (0.46–0.64)	13.52 ± 1.98 (10.50–18.01)
**Hind limb, left/L**	SJD	19.89 ± 1.46 (17.45–21.58)	51.15 ± 8.45 (36.90–60.89)	17.73 ± 2.30 (13.48–20.16)	0.43 ± 0.11 (0.59–0.30)	0.46 ± 0.06 ^a^ (0.38–0.53)	11.80 ± 1.80 (9.52–14.53)
	FHO	19.91 ± 1.33 (17.65–22.41)	47.30 ± 6.85 (38.48–61.48)	18.13 ± 1.41 (15.95–20.87)	0.48 ± 0.10 (0.34–0.66)	0.51 ± 0.04 ^a^ (0.44–0.58)	12.57 ± 2.01 (9.58–16.97)
**Hind limb, right/CO**	SJD	21.41 ± 1.62 (19.15–23.38)	52.09 ± 7.83^a^ (36.10–58.71)	22.00 ± 2.22 (18.13–25.32)	0.48 ± 0.11 (0.36–0.66)	0.45 ± 0.04 ^a^ (0.40–0.53)	12.03 ± 1.67 (10.33–14.17)
	FHO	20.61 ± 1.39 (18.10–22.74)	43.47 ± 6.09^a^ (35.48–55.71)	21.14 ± 1.42 (19.08–24.17)	0.52 ± 0.11 (0.38–0.72)	0.50 ± 0.05 ^a^ (0.39–0.57)	12.74 ± 2.10 (9.86–17.48)

CO = contralateral, DI = diagonal, FHO = cats after femoral head and neck ostectomy, IFz = vertical impulse, IPS = ipsilateral, L = lame, PCA = paw contact area, PFz = peak vertical force, SJD = cats with stifle joint disease, SL = step length, SPD = stance phase duration, StPh = stance phase, TF = total force, TPFz = time to PFz. The grey shaded area indicates the lame leg.

^a^Significant difference for the leg (p < 0.05)

**Fig 1:**
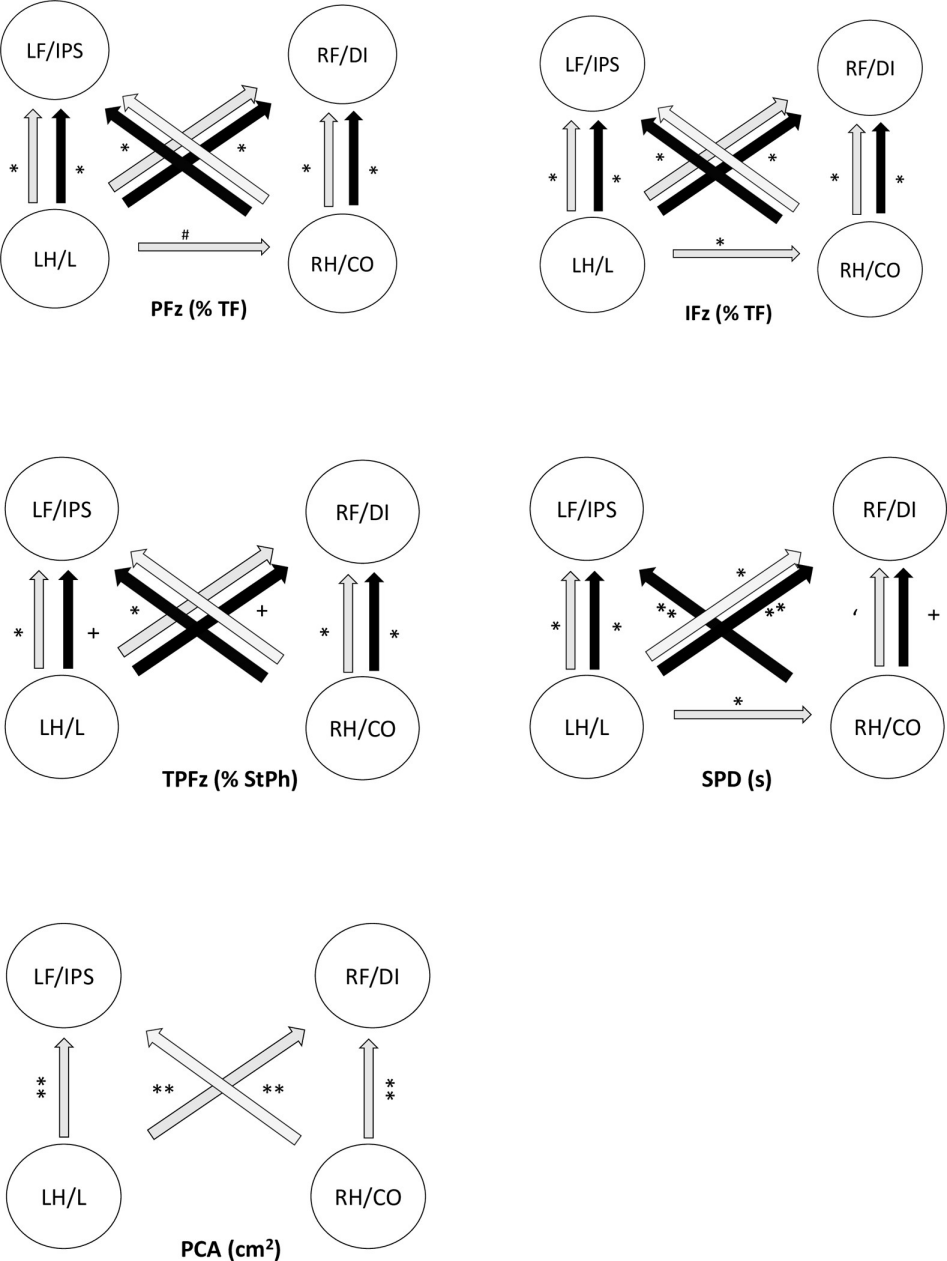
Statistically significant differences in the limbs between the LC and NLC groups. The arrows are pointing towards the higher values. The grey shaded arrows indicate the LC and the black arrows indicate the NLC. *p = 0.000, **p = 0.001, ‘p = 0.002, ^+^p = 0.003, ^#^p = 0.036, CO = contralateral, DI = diagonal, IFz = vertical impulse, IPS = ipsilateral, L = lame, LC = lame cats, NLC = sound cats, PCA = paw contact area, PFz = peak vertical force, SL = step length, SPD = stance phase duration, StPh = stance phase, TF = total force, TPFz = time to PFz.

There was a statistically significant difference (p < 0.036 and p = 0.001) in the PFz (% TF) and IFz (% TF) measurements between the lame limb and the contralateral, ipsilateral, and diagonal limbs in the LC group. Furthermore, there were significant differences between the contralateral limb and the diagonal limb and between the contralateral limb and the ipsilateral limb (both p = 0.000) but there was no difference between the ipsilateral and diagonal forelimbs in the LC group. In the control group, there was a statistically significant difference in the PFz and IFz between the forelimbs and hind limbs (both p < 0.001, Fig 1). There was no significant difference in GRFs in the LC group according to whether the disease affected the stifle joint or the cats had a FHO. Comparing the LC and NLC groups, the general linear model revealed a significant effect (p = 0.008) of leg and group (lame vs. non-lame) for IFz (% TF). Specifically, the IFz (% TF) was significant lower in the affected limb in the LC group than in the reference leg in the NLC group (p = 0.001).

### Symmetry index

In the LC group, the SI for the PFz was 1.69 ± 1.48% in the forelimbs and 3.61 ± 2.27% in the hind limbs and was 3.84 ± 2.64% and 8.81 ± 3.67%, respectively, for the IFz; the respective values in the NLC group were 1.35 ± 0.89% and 3.14 ± 1.79% for the PFz and 1.86 ± 1.39% and 2.40 ± 2.34% for the IFz. The SI values for the PFz and IFz were significantly higher in the hind limbs than in the forelimbs in the LC group (p = 0.001 and p = 0.000, respectively). There was no significant difference in the SI for the PFz between the LC and NLC groups; however, the SI for the IFz was always significantly higher in the forelimbs (p = 0.013) and hind limbs (p = 0.000) in the LC group then in the NLC group. There was no significant difference in the SI according to whether the problem was in the stifle or after FHO in the LC group.

### Time to PFz

In the LC group, the time to PFz was significantly later in the ipsilateral and diagonal forelimbs than in the lame and contralateral hind limbs (p = 0.000). There was no significant difference in the time to PFz between the forelimbs or between the hind limbs. The time to PFz was also significantly longer in the forelimbs than in the hind limbs in the NLC group (p < 0.003). In the LC group, the time to PFz was significantly longer in the contralateral limb in cats with a stifle problem than in those after FHO (p = 0.014). There was a significant difference in time to PFz for the diagonal limb (p = 0.011) between the LC and NLC groups; it was longer in the LC group (Fig 1).

### Stance phase duration

In the LC group, the SPD was significantly shorter in the lame limb than in the contralateral, ipsilateral, and diagonal limbs (p = 0.000). However, there was no significant difference in SPD between the ipsilateral forelimb and the contralateral hind limb or between the ipsilateral and diagonal forelimb (Fig 1). In the NLC group, the SPD was significantly longer in the forelimbs than in the hind limbs (p < 0.003).

There was no statistically significant difference in SPD between cats in the LC group according to whether the stifle joint was affected or after FHO. However, in the general linear model, a significant difference between the LC and NLC groups regarding SPD was noted. The SPD was longer in the contralateral limb (p = 0.015), ipsilateral limb (p = 0.025), and diagonal limb (p = 0.010) but not in the lame limb in the LC group when compared with the NLC group.

### Step length

The mean step length was 0.49 ± 0.05 m in the LC group and 0.50 ± 0.05 m in the NLC group. There was no difference in step length between the LC and NLC groups or between the limbs. However, in the LC group, the step length was significantly shorter for all four limbs when the stifle joint was affected (lame limb, p = 0.038; contralateral limb, p = 0.036; ipsilateral limb, p = 0.041; diagonal limb, p = 0.005) than after FHO.

### Paw contact area

There was no significant difference in mean PCA between the LC group and the NLC group (12.82 ± 1.81 cm^2^ vs. 12.49 ± 1.87 cm^2^). However, the PCA was significantly larger in the forelimbs (ipsilateral and diagonal) than in the hind limbs (lame and contralateral) in the LC group (p< 0.003). In the LC group, there was no significant difference between the forelimbs and hind limbs (Fig 1) according to whether the stifle joint was affected or after FHO.

## Discussion

In this study, we compared a group of lame cats with a group of sound cats and confirmed that lame cats have changes in GRFs and temporospatial parameters in the hindlimb that can be detected by a pressure plate at a walk. This finding confirmed the first part of our hypothesis, i.e., that the IFz (% TF) and PFz (% TF) are lower in the lame limb than in the contralateral limb in lame cats; however, the difference between lame and sound was significant for IFz (% TF) but not for PFz (% TF). We were not able to confirm the second part of our hypothesis. The GRFs measured were comparable in cats with stifle disease or lameness after FHO.

Peak vertical force and vertical impulse are the most commonly used descriptors of normal and pathologic gait [26]. Vertical forces mainly measure weight-bearing and have the greatest magnitude when compared with other orthogonal forces. Those vertical forces are lower than the normal reference levels when lameness is present [26]. Studies have established that normalization of PFz and IFz to the total force (% TF) is adequate and reliable for describing gait in cats and dogs [9, 10, 25]. In particular, the coefficient of variation for IFz is more stable when normalized to TF. Therefore, we normalized it to TF in our research and not to % BM, as is often the case [6 –8, 12–15, 20–24].

A careful review of the literature showed that PFz and IFz could detect lameness and evaluate the results of treatment in cats [12–15, 20, 21], which is consistent with our present finding of a lower PFz (% TF) and IFz (% TF) in the lame limb than in the other three limbs. However, in our study, only the IFz (% TF) and SPD could differentiate between a lame limb and a sound one.

Lameness in dogs has been studied more extensively [27–34]. Compensatory mechanisms have been demonstrated for forelimb [27–29] and hind limb [30–34] lameness in canines. Dogs will shift the load mainly to the contralateral hind limb and not to the forelimbs when attempting to unload an injured hind limb. Therefore, it was concluded that the contralateral hind limb should not be used for control purposes [35, 36]. Another report described compensatory changes in the diagonal forelimb because of hind limb lameness [33]. In contrast, we found no increase in force in the contralateral hind limb between lame and sound cats. Furthermore, there was no significant increase in forces in the forelimb. Therefore, it seems that cats compensate for moderate hind limb lameness by redistributing these forces equally to all other three limbs, without favoring either the contralateral, ipsilateral, or the diagonal sound limb. This finding suggests that the contralateral limb can be used as the control in cats with moderate hind limb lameness when assessing the outcomes after surgery.

It is widely believed that gait analysis can be used to differentiate lame and sound animals. However, a study has reported that 15 of 41 dogs with lameness could not be discriminated from normal dogs at a walk. Three of these dogs even had an SI within the reference range [31]. Investigators in previous studies confirmed that the calculated SI was similar in magnitude between cats and dogs [7, 10, 37]. In our study, we were able to confirm lameness when the SI for the IFz was higher in the hind limbs than in the forelimbs. Furthermore, the SI was higher in both the forelimbs and hind limbs in lame cats than in sound cats, indicating that the gait is more asymmetric overall in lame cats.

Similar to dogs [25, 37–40], in our study, the PFz (% StPh) was greater in the forelimbs of the cats than in the hind limbs. The lame cats in our study showed an additional feature, i.e., that the time to PFz (% StPh) was delayed in the diagonal limb in comparison with the diagonal limb in a sound cat. This finding might reflect compensation by the diagonal forelimb, which has been described in dogs [33]. However, we could not confirm this part of our hypothesis because we did not measure different GRFs.

Changes in SPD are common in lame dogs, which show an increase in SPD in the diagonal forelimb and contralateral hind limb, and a decrease in the ipsilateral forelimb, during walking [33]. In our study, the SPD was significantly higher in all three other limbs in the lame cats than in the sound cats. Again, this might be a sign of the weight being shifted onto the other limbs, thereby compensating with the contralateral, ipsilateral, and diagonal limbs. This finding could also reflect a difference in velocity, although the velocity in our two study groups was in the range of 0.30–0.85 m/s with no significant between-group difference, which is comparable with the velocities of 0.30–1.7 m/s in other reports [9]. Therefore, we excluded different velocities as a cause for the difference in SPD.

The paw contact area in our lame cats was larger in the forelimb than in the hind limb. As already mentioned, the reason for this finding was the change in forces exerted on the forelimb. Whether or not this applies to all lame cats needs to be confirmed by measurement of the center of pressure in further studies.

Canine stifle osteoarthritis has more impact on gait function than hip osteoarthritis, resulting in a greater reduction in GRFs [32]. In a study by Stadigh et al in 2016 [23], cats with cranial cruciate disease and stifle osteoarthritis showed a higher SI for PFz but not for IFz in the forelimb and hind limbs. Furthermore, the authors demonstrated a redistribution of weight from the affected to the unaffected limbs and a decreased PFz and IFz for the lame limb. The reasons for the decrease in forces are likely to include instability of the stifle joint, muscle atrophy, altered proprioception, and a modified gait pattern to avoid or minimize pain [23].

In our study, the PFz and IFz (% TF) in cats with stifle disease were lower than those in all other limbs were but not different from those in cats after a FHO has been performed more than one year ago. Therefore, we could not confirm that lameness due to stifle disease is more painful than lameness due to a previous FHO in cats. However, the time to PFz (% StPh) was longer in the contralateral hind limb in cats with a stifle problem than in those after FHO. One reason for this could be that propelling the limb forward after the stance phase is more painful in cats with a stifle problem or less painful in cats after FHO due to the elevation of pain in the hip joint.

We were also not able to detect a difference in SPD in cats according to whether the lameness was attributable to a problem in the stifle or the hip. Controversially, there was a significant difference in step length between cats with stifle lameness and those with a lameness after FHO. These cats had a shorter stride in all limbs. Even if the velocity was the same in both groups, it is possible that cats with stifle disease shift their weight from one limb to another more rapidly and take more steps than cats after FHO. However, we could not demonstrate an increased impact of stifle disease on the limb, and further investigations in larger study populations are needed. It would be useful to include kinematics when investigating the changes in gait in cats with stifle or hip joint problems. Moreover, a recent study has shown that three-dimensional kinematics can also be used when evaluating joint angles in cats. A multiplanar model has the potential to capture clinically relevant changes in kinematics in detail and improve our understanding of kinematic motion beyond what can be determined by sagittal and composite sagittal-frontal plane techniques. This model would also create an opportunity to determine the amount of internal rotation of the tibia in relation to the femur in cruciate-deficient stifles [41].

Gait analysis seems to be the gold standard for detection of lameness in dogs [1, 3, 26–30, 32–36]. Nevertheless, studies have shown that not all dogs clinically assumed to be lame show changes on the force plate [31, 42]. The study investigators hypothesized that whole-body movement is not accounted for when measuring GRFs, such that clinically lame dogs can have normal GRFs. In contrast, we found that five cats were not lame during visual gait analysis; these cats displayed reduced GRFs on the plate and had a history of a pathologic disease requiring unilateral FHO. We believe that the difficulties encountered when examining cats in the consultation room stem from their inherent anxiety and not being able to walk normally in a stressful situation. It is likely that the results would have been different if videos of the cats walking freely at home had been obtained.

This study has some limitations. First, we only included cats with mild to moderate lameness, and it is possible that the total forces on the contralateral hind limb would be greater in cats with more severe lameness. Furthermore, all cats were assessed at a walk and not at a trot, and canine studies have shown that low-grade lameness might be better measured at a trot [31]. Whether or not this applies to a cat is unknown. It is possible that our assessment technique was unable to detect subclinical lameness and further studies are needed to measure lameness in cats while at a trot. The sample size in cats with stifle disease and after FHO is small. Difficulties in comparing those two groups might also arise from the different stifle conditions, which are included. Therefore, these results have to be interpreted carefully. Furthermore, it is yet unknown whether there is a difference in compensatory mechanisms (e.g., increased forces in the contralateral limb) between dogs and cats.

## Conclusion

We have demonstrated that mild to moderate lameness that cannot be detected by visual gait analysis on orthopedic examination can be detected in cats walking on a pressure plate. Cats seem to compensate for lameness by distributing the force to all other limbs and not exclusively to the contralateral or diagonal forelimb like in dogs. In this study, we were less able to detect major changes in GRFs in cats with stifle than in those after FHO. Further three-dimensional kinematic studies in larger populations of cats are needed to characterize lameness due to different pathologic conditions in more detail.

## Supporting information

S1 Dataset Data of the 20 lame and 15 sound cats. (XLSX)
